# Artificial Intelligence for Identifying Patient-Reported Outcome and Experience Measures in Oncology: Retrospective Cross-Sectional Study Using ClinicalTrials.gov

**DOI:** 10.2196/84533

**Published:** 2026-04-16

**Authors:** Jessica Soyer, Akram Hecini, Sylvain Juchet, Céline Desvignes-Gleizes, Maxime Thiebaut, Jean-Philippe Bertocchio

**Affiliations:** 1 SKEZI Annecy France; 2 Mapi Research Trust Lyon France; 3 Thyroïde – Tumeurs Endocrines, Pitié-Salpêtrière Hospital Assistance Publique-Hôpitaux de Paris Paris France; 4 Reference center for rare diseases related to calcium-phosphate metabolism disorders, Pitié-Salpêtrière Hospital Assistance Publique-Hôpitaux de Paris Paris France; 5 FHU-DDS-net, Dental School Université Paris Cité, INSERM U1333 Santé Orale Montrouge France

**Keywords:** clinical trials, patient-reported outcome measures, patient-reported experience measures, artificial intelligence-based research, expert-based research, oncology, research on research

## Abstract

**Background:**

Traditional health care systems have evolved to increasingly recognize patients’ perspectives as key to improving the quality of care, especially in oncology. Hence, patient-reported outcome measures (PROMs) and patient-reported experience measures (PREMs) can enhance patient–health care provider communication while facilitating individualized care. This tailored approach improves patient outcomes and highlights the need for research methods to account for variability in patient experiences across diverse sociodemographic and clinical backgrounds while proposing artificial intelligence (AI) to automate and accelerate the identification of PROMs and PREMs.

**Objective:**

This study aimed to estimate the proportion of clinical studies including PROMs or PREMs, using either a traditional expert-based identification method or an AI-enriched approach.

**Methods:**

In a retrospective cross-sectional study using the ClinicalTrial.gov database, we focused on oncology studies between 2012 and 2022. Two methods were used to identify PROMs and PREMs: (1) a traditional expert-based method, where an algorithm identified PROMs and PREMs from a list of 346 oncology-specific PROMs and PREMs (extracted from the PROQOLID database, Mapi Research Trust) and/or 11 PROM- and PREM-specific terms, and (2) an AI-enriched method using a bidirectional encoder representations from transformers model, trained on 2399 outcomes labeled by experts. To evaluate algorithm performance, results were compared with expert decisions. Studies were classified by PROM/PREM use, analyzed using logistic regression to identify drivers, and a named entity recognition model identified frequently used measures.

**Results:**

A total of 24,491 studies were included. According to the traditional expert-based algorithm, 7549 (31%) studies used at least one PROM or PREM, as compared to 8029 (33%) studies identified by the AI-enriched algorithm, increasing from 2012 to 2022 (*P*<.001). With 90% (95% CI 88%-92%) accuracy, the AI-enriched algorithm outperformed (*P*<.001) the expert-based algorithm (84%, 95% CI 82%-86%) in identifying PROMs and PREMs. Breast and digestive cancers accounted for nearly 50% of all oncology studies using PROMs and PREMs, with the European Organization for Research and Treatment of Cancer Quality of Life Questionnaire-Core 30 being the most frequently used. As expected, trials in phases 2 to 4 more frequently included PROMs and PREMs than preclinical or early phase 1 studies (odds ratio [OR] 1.8, 95% CI 1.1-2.8 for phase 2; OR 3.6, 95% CI 2.3-5.8 for phase 3; and OR 2.6, 95% CI 1.6-4.4 for phase 4). In observational studies, cross-sectional and prospective studies incorporated more PROMs and PREMs than retrospective studies (OR 4.6, 95% CI 3.3-6.4 and OR 3.2, 95% CI 2.5-4.1, respectively).

**Conclusions:**

Our study shows that an AI-enriched algorithm outperforms traditional expert methods in identifying PROMs and PREMs in oncology. Combining expert-labeled data with AI enables scalable, automated trial monitoring, supports efficient research, informs stakeholders, enhances patient-centered decisions, and can be extended to other diseases and databases.

## Introduction

Traditional models in health care have evolved to increasingly recognize patients’ perspectives as key to improving their quality of services [1]. In such, the quality of data is of major importance for evaluating services provided to people. To date, most of the data to do so comes from automatically generated sources emerging from health medical records and related software. Besides, data collected from patient-centered measures (PCMs) may also play a key role in improving the quality of services [2]. PCMs refer to widely used and studied tools, such as patient-reported outcome measures (PROMs) or patient-reported experience measures (PREMs) [3], which constitute relevant information collected directly from patients to better understand and address what matters the most to them [2,4]. In their day-to-day practice, health care providers (HCPs) may collect PCMs for various purposes such as screening, monitoring, evaluating care, or collecting side effects of treatments as well as to help in the decision-making process [5]. PCMs are usually collected through questionnaires (either paper-based or online) and help capture either outcomes resulting from the care (such as the evolution of symptoms) or the experience of such care (such as the time spent receiving a specific treatment). Items (questions) can be scientifically validated, and therefore, such questionnaires are called PROMs or PREMs for collecting outcomes or experiences, respectively, or made for the purpose of the questionnaire itself, and are therefore called *ad hoc* items. These measurements are added to biological measures and/or clinician-reported outcomes, such as physical examination, to better plan patient care.

PROMs are standardized questionnaires that collect information about health outcomes directly from patients, such as symptoms, functional status, health-related quality of life (HRQoL), or mental well-being, without amendment or interpretation by a clinician or anyone else, and measure them in a quantitative manner at a specific timepoint [6-8]. Originally developed in research studies, PROMs applications are now diversified and used in supporting clinical decision-making, assessing treatment benefit in drug trials, prioritizing patients for surgical procedures, comparing outcomes among HCPs, stimulating HRQoL improvement, and evaluating practices [9-11]. There are two main categories of PROMs: (1) generic and (2) specific ones (including both condition-specific and concept-specific items). Generic PROMs measure concepts relevant to a wide range of patients, enabling comparisons across different conditions. For example, the EQ-5D questionnaire [12,13] measures 5 dimensions of health: mobility, self-care, usual activities, pain or discomfort, and anxiety or depression. Each dimension is assessed using a set of severity levels, initially comprising 3 levels (EQ-5D-3L) and expanded to 5 levels in the updated 2010 version (EQ-5D-5L), notably to improve sensitivity and reduce ceiling effect [13]. Specific PROMs, on the other hand, measure concepts relevant to a particular patient group or condition. For instance, the European Organization for Research and Treatment of Cancer Quality of Life Questionnaire-Core 30 (EORTC QLQ-C30) questionnaire measures HRQoL specifically in patients with cancer (condition-specific) [14], while the Fatigue Severity Scale (FSS) evaluates the concept of fatigue (concept-specific) [15]. Besides this core common questionnaire for all patients with cancer, additional modules have been developed from it to measure symptoms and outcomes specific to different cancers. Examples include the EORTC Quality of Life Questionnaire–Lung Cancer Module (EORTC QLQ-LC13) or the Head and Neck Cancer Module (EORTC QLQ-H&N35) [16,17]. For the sole field of cancer, more than 300 PROMs have already been identified.

In contrast to PROMs, PREMs focus on patients’ experience and emerge as a favored metric for assessing health care quality, such as the validated Kidney PREM questionnaire [18]. PREMs assess common information about patients satisfaction with service delivery in a clinical setting or describe patients experiences with a therapy or a plan of care [4,8]. Like the US Food and Drug Administration (FDA), other public health authorities in Europe, such as the Haute Autorité de Santé (HAS) in France, have recently added PROMs and PREMs to their list of care quality and safety indicators to encourage their use in clinical trials and routine [8,19-21]. However, there is no established consensus regarding the way to name PROMs and PREMs. This makes their identification particularly challenging. New tools based on natural language processing (NLP) may be useful in this setting [22]. Overall, it appears essential to develop a solution for identifying and tracking the use of PROMs and PREMs in clinical research, both in terms of their frequency of inclusion in studies and the types of instruments selected. We hypothesized that an artificial intelligence (AI)–enriched algorithm would outperform an expert-based algorithm in identifying PROMs and PREMs used in clinical research. Here, we report the results from a cross-sectional study that aimed to estimate the proportion of oncology clinical studies incorporating PROMs and PREMs and to examine the characteristics associated with these studies, using either a traditional expert-based method or an AI-enriched approach. The performances of the expert-based and AI-enriched algorithms were evaluated through comparison to a manually labeled subsample validated by a human-based consensus.

## Methods

### Study Design and Data Sources (Setting)

Our retrospective cross-sectional study uses data regarding outcomes of interest from previously documented clinical studies. We conducted a retrospective cross-sectional study to provide a comprehensive assessment of the prevalence and characteristics of PROMs and PREMs in oncology trials over a defined period (2012-2022). This design was chosen because all study data were already publicly available in the ClinicalTrials.gov registry, allowing us to analyze a large dataset without prospectively collecting new data, and to efficiently capture a snapshot of PROMs and PREMs inclusion across multiple studies at a single point in time. This data was extracted from the ClinicalTrials.gov database, more specifically, the “What is the study measuring?” section of the studies’ information page, using the *trials* R package. To be included, studies had (1) to be available in the open access ClinicalTrials.gov database; (2) to have a study start date between January 1, 2012, and January 1, 2022; (3) to be set in the oncology field; and (4) to contain at least one of our described outcomes. Studies that contained no outcome description were therefore excluded. If one study had more than one outcome, all available outcomes were extracted individually. Because the study relied exclusively on publicly available registry data, nonparticipation was not applicable.

### Identifying PROMs and PREMs

#### Overview

Two different techniques were used to identify PROMs and PREMs in oncology studies’ outcomes: (1) an expert-based algorithm and (2) an AI-enriched algorithm. In addition, a human-based consensus manually labeled (as containing or not PROMs or PREMs) a subset of the included outcomes. This human-based consensus was used first to feed the AI-enriched algorithm and act as a gold standard for the evaluation of the performance of the two algorithms.

#### Expert-Based Algorithm

A list of oncology-specific PROMs and PREMs and PROM- and PREM-related generic open terms was gathered. First, 346 PROMs and PREMs used in oncology, including both their full names and acronyms, were extracted from the Patient-Reported Outcome and Quality of Life Instruments Database (PROQOLID). Second, 2 distinct groups of 3 experts set up a list of terms related to generic PROMs and PREMs for the purpose of this study. The experts then reached a consensus, resulting in a final list of PROM- and PREM-related generic open terms. PROM-specific terms were* *quality of life, QoLd, patient-reported outcome, psychometric, patient perspective, questionnaire, health related quality of life, and hrql. PREM-specific terms were satisfaction, experience, and quality of care.

The list of 346 oncology-specific PROMs and PREMs and 11 PROM- and PREM-related generic open terms was then used in the expert-based algorithm. To identify outcomes, including PROMs and/or PREMs, the expert-based algorithm specifically searched for PROMs or PREMs and specific terms within the outcome descriptions available in the dataset of oncology studies extracted from ClinicalTrials.gov.

#### Expert Consensus

A subset of studies’ outcomes was randomly selected to be manually labeled by 16 independent experts (ie, not involved in the process of identifying the PROMs and PREMs generic terms). This random subset was built to balance positive and negative outcomes, that is, included 15% of the outcomes with PROMs or PREMs as identified by the expert-based algorithm and 2% of the outcomes without PROMs or PREMs. The 16 experts were divided into 8 pairs; each pair was assigned a random sample of the subset to label. Experts were asked to label whether or not the descriptions contained either a PROM or a PREM: to be counted as mentioning a PROM or PREM, a description should provide a name (either an acronym or a full name) referring to a questionnaire that should be answered directly by patients and that should assess either an outcome or an experience of care. Therefore, we excluded descriptions that mentioned using a PROM or PREM but that did not state which one, that used an ad hoc questionnaire, or that used a clinician-reported outcome measure. While labeling the outcomes, experts were blind to the identity of their pairs and to the label provided by their pairs. Once all outcomes were labeled, experts were unblinded to their pair and reached consensus in case of disagreement (Cohen κ 0.59), setting the gold-standard label for the outcome. During the whole process, experts were blind to the classification according to the expert-based algorithm. Even if the initial agreement between the 2 experts was 59% only, this first round was only a starting point. For all cases where disagreements occurred, the experts engaged in detailed discussions and conducted additional reviews of the scientific literature to resolve them until a consensus was reached. Through this consensus-building process, all disagreements were carefully examined and validated, leading to final labels that are 100% agreed on by both experts and grounded in verified evidence. Therefore, while the initial agreement reflects the complexity of the task, the final gold standard used for evaluation is fully consensual and reliable.

#### AI-Enriched Algorithm

The AI-enriched algorithm consisted of a text classification model built using bidirectional encoder representations from transformers (BERT) [23]. This model used texts from outcomes descriptions labeled by the expert consensus as containing or not PROMs or PREMs. For the model requiring balanced classes (ie, equal number of outcomes with and without PROMs or PREMs), a corresponding subset of the manually labeled outcomes was used. The dataset was split as follows: 61% of the data were used for training (2399 samples), 12% for validation (479 samples), and 27% for testing (1065 samples). Using BERT Tokenizer [23], each word or textual element was converted into a token, which was then encoded as a fixed-length numerical vector of 512. This numerical vector captured semantic and contextual information within the texts. Model training was conducted using the Trainer from the Transformers library developed by Hugging Face [24]. During training, multiple models were generated by adapting batch size and learning rate to get the best performance. All generated models were evaluated using the associated validation subset. The performance of each model was assessed using the following metrics: accuracy, recall, and *F*_1_-score. These metrics gauged the model’s ability to correctly classify texts into their respective categories. The best model was selected based on its accuracy score.

### Evaluating the Performance of Algorithms

To evaluate algorithms’ performances (accuracy, sensitivity, and specificity), the results obtained by expert-based and AI-enriched algorithms were compared to a dataset of outcomes labeled manually by expert consensus (gold standard). Evaluations were performed on the same dataset corresponding to the performance evaluation subset randomly selected when building datasets for the AI-enriched algorithm.

### Describing the Studies Using PROMs and PREMs

Each study was classified as using at least one PROM or PREM or not, based on an AI-enriched algorithm (or the expert-based algorithm, see Multimedia Appendix 1). As observational and interventional studies carried out different characteristics, the analyses were stratified according to the type of study. We used multivariate logistic regression analysis to investigate potential associations between the use of PROMs and PREMs adjusted to other characteristics: cancer site, intervention type, clinical trial phase, randomization type, model type, and time perspective.

### Recoding Cancer Sites

The ClinicalTrials.gov database returns a wide variety of names for cancer sites, even for the same cancers, as this field is an open-label one. Cancer sites had to be recoded in order to enhance the homogeneity and accuracy of the data. First, a *k*-means clustering [25] was used to group the cancer sites based on the similarities between names for each clinical study. The 10 most recurrent words in each cluster were identified for naming each cluster. The cluster names (cancer sites) were then used in a subsequent few-shot classification task, using the setfit model from Hugging Face [26]. For each cancer site, eight representative sentences were carefully selected from the dataset, which were used to create a final set of 116 texts. This dataset was used to train a text classification model, which was then applied to classify all the cancer sites in the dataset. The final model presented an accuracy of 96% for classifying cancer sites.

### Identifying the Most Frequently Used PROMs and PREMs

Although the main AI-enriched algorithm was designed to detect if a PROM or PREM was used or not, it was not designed to state which PROM or PREM it was referring to. To identify which specific PROMs or PREMs were the most frequently used in clinical research, we used a named entity recognition (NER) model called SKEP’IA, which we had previously developed and patented. First, we manually annotated more than 2500 outcomes containing PROM names using the open-source annotation tool Doccano. Second, using the open-source library for natural language processing in Python, spaCy, these entities were then converted into a BIO tagging scheme (beginning, inside, outside) to mark the start, continuation, and boundaries of PROM entities within the text. The NER model was trained on this structured dataset. Third, we fine-tuned a BERT-cased model, splitting the dataset into training, validation, and test sets using appropriate proportions. Hyperparameters were optimized with Optuna. Fourth, the final model was evaluated on the test set. This model can recognize any PROM or PREM within a text, whether they are presented in full text or as an acronym and can suggest relevant ones based on textual descriptions. SKEP’IA was developed by using labeled data collected from both ClinicalTrials.gov and PubMed. However, the absence of a standardized and official naming system for each PROM or PREM presented a challenge in determining the most commonly used ones, representing a limitation in the exclusive use of the SKEP’IA model. To address this issue, we implemented a text similarity search approach using the PROQOLID database. Thus, the PROMs and PREMs identified by SKEP’IA were compared to the list of PROMs and PREMs previously captured from the PROQOLID database by calculating text similarity between them. Finally, we combined both the NER model and the text similarity search in the PROQOLID database.

### Statistical Analysis

Statistical analyses were performed using R Studio software (version 4.4.2; Posit PBC). Descriptive statistics were used to illustrate the use of PROMs and PREMs in registered clinical studies. Characteristics of the studies were presented by their numbers and proportions. Continuous variables were analyzed as reported in the registry and were not categorized. Proportions were compared bivariately using a chi-square test. Missing data were reported where appropriate in the descriptive table, and their randomness was assessed using a missing completely at random (MCAR) test (RBtest R package) with results displayed in the table footnote. Year-over-year trends in the use of PROMs and PREMs were described. A multivariate logistic regression analysis was used to investigate possible associations between the use of PROMs and PREMs and study characteristics, by subgroups of interventional or observational studies. The variables included in the models were those obtained from the ClinicalTrials.gov database and considered relevant to the study. The odds ratios (ORs) were presented using forest plots, and statistical significance for each predictor was assessed using Wald tests. Because associations were expressed as ORs, translation into absolute risk was not applicable. A *P* value less than .05 was considered as reflecting statistical significance. No subgroup, interaction, or sensitivity analyses were prespecified or conducted, as the study followed a predefined analytical framework.

### Ethical Considerations

This study did not involve human participants or the use of identifiable human data. Therefore, institutional review board approval, informed consent, and participant remuneration were not applicable. Our observational study is compliant with the STROBE (Strengthening the Reporting of Observational Studies in Epidemiology) guidelines (checklist in Multimedia Appendix 1). No images or figures in the manuscript or multimedia appendix contain information that could identify individual participants or users.

## Results

### From the Selection of Studies to PROMs and PREMs Identification

**Figure 1:**
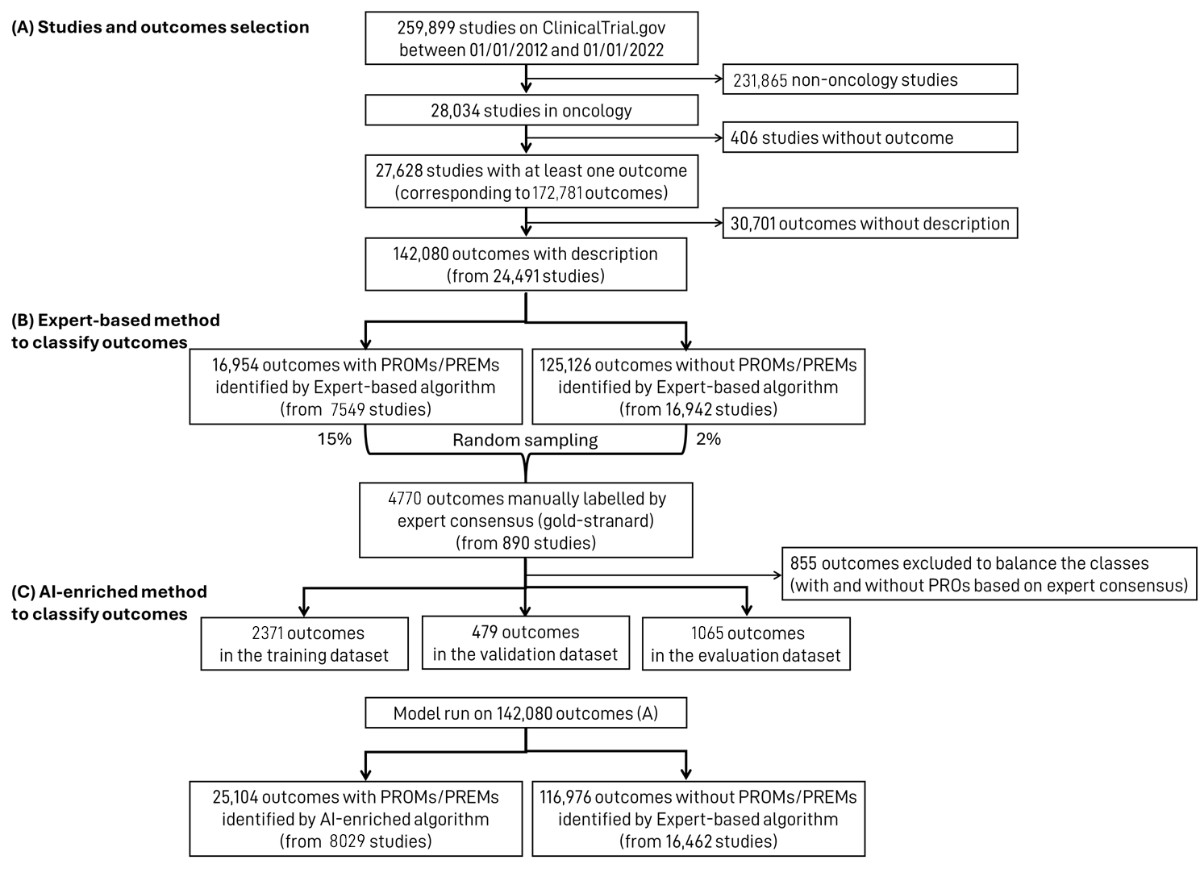
Flowchart displaying studies and outcomes selection (A), the expert-based (B) and artificial intelligence (AI)–enriched (C) methods to classify outcomes, ClinicalTrial.gov database, 2012-2022. (A) From 259,899 studies registered between January 1, 2012, and January 1, 2022, a total of 28,034 oncology studies were identified. After excluding studies with no outcomes and outcomes lacking descriptions, 142,080 outcomes (from 24,491 studies) were included. (B) An expert-based method identified 16,954 outcomes using patient-reported outcomes measures (PROMs) or patient-reported experience measures (PREMs) across 7549 studies. A random sample of 4770 outcomes (890 studies) was manually labelled by expert consensus (15% of PROMs- or PREMs-positive and 2% of PROMs- or PREMs-negative outcomes). (C) The expert consensus dataset was then used to train and evaluate an AI-enriched algorithm, which was then applied to the full dataset of 142,080 outcomes. The AI-based algorithm identified 25,104 PROMs or PREMs outcomes across 8029 studies.

Between January 1, 2012, and January 1, 2022, a total of 28,034 studies in oncology were found in the ClinicalTrials.gov database, including 24,491 with at least one outcome described (Figure 1A). According to the expert-based algorithm, 16,954 outcomes using PROMs or PREMs were found from 7549 studies, meaning that out of the 24,491 eligible oncology studies, 31% used at least one PROM or PREM (Figure 1B). A subset of 4770 studies’ outcomes was used to evaluate the performance of the expert-based algorithm. When compared to the expert consensus manually labeled gold standard, the expert-based algorithm achieved 83% (95% CI 82%-84%) accuracy, 86% (95% CI 85%-88%) sensitivity, and 81% (95% CI 80%-82%) specificity (Table 1).

**Table 1:** Confusion matrix comparing the identification of patient-reported outcome measures (PROMs) or patient-reported experience measures (PREMs) in the outcomes of oncology studies, determined by either expert-based or artificial intelligence (AI)–enriched algorithms vs expert consensus, ClinicalTrials.gov database, 2012-2022 (n=1065 outcomes in the test subset)^a^.

Type of algorithm and use of PROMs or PREMs in the study outcome		Expert consensus			PPV^b^ (%)		NPV^c^ (%)
		Yes (n=506)	No (n=559)				
**Expert-based algorithm**					80		88
	Yes (n=558)	448	110				
	No (n=507)	58	449				
**AI-enriched algorithm**					88		92
	Yes (n=520)	460	60				
	No (n=545)	46	499				

^a^This table shows the classification performances of the expert-based and AI-enriched models in predicting if study outcomes included PROMs or PREMs, compared to the expert consensus gold standard. “Yes” and “No” refer to whether outcomes included PROMs or PREMs. The expert-based algorithm achieved an accuracy of 84% (95% CI 82-86), a sensitivity of 89% (95% CI 85-91), and a specificity of 80% (95% CI 79-85), yielding a PPV of 80% and an NPV of 88%. The AI-enriched algorithm achieved an accuracy of 90% (95% CI 88-92), a sensitivity of 91% (95% CI 88-93), and a specificity of 89% (95% CI 86-92), yielding a PPV of 88% and an NPV of 92%.

^b^PPV: positive predictive value.

^c^NPV: negative predictive value.

The AI-enriched algorithm identified 8029 studies that used at least one PROM or PREM, representing 33% (Figure 1C). In the performance evaluation subset of 1065 outcomes, using the expert consensus manually labeled gold standard, the AI-enriched algorithm achieved 90% (95% CI 88%-92%) accuracy, 91% (95% CI 88%-93%) sensitivity, and 89% (95% CI 86%-92%) specificity (Table 1). Therefore, the AI-enriched algorithm performed better (chi-square test *P*<.001) than the expert-based algorithm in identifying PROMs and PREMs in oncology studies. The following results present the characteristics of studies identified based on the AI-enriched algorithm only. The results obtained using the expert-based algorithm are available in Multimedia Appendix 1.

### Trends in PROMs and PREMs Use in Oncology Studies Over Time

The analysis of year-over-year trends revealed a steady increase in the proportion of clinical studies incorporating one or more PROMs or PREMs, from 27.6% in 2012 to 36.2% in 2021 (Figure S2 in Multimedia Appendix 1).

### Characteristics of Studies

Table 2 summarizes the characteristics of the 24,491 studies included in the analysis, stratified on whether they used PROMs or PREMs (n=8029) or not (n=16,462), as identified by the AI-enriched algorithm. Among all the included studies (n=24,491), 79% (n=19,344) were interventional trials.

Focusing on these interventional trials, phase 2 (n=5228, 27%) was the most common study stage, followed by phase 1 (n=2536, 13.1%), phase 3 (n=1650, 8.5%), and phase 1/2 (n=1528, 7.9%) to report using PROMs or PREMs. However, missing data regarding trial phases accounted for 37.7% (n=7285) of studies (MCAR test indicated missing at random). The most frequent allocation type was randomization, accounting for 44.4% (n=8586) of interventional studies. The predominant intervention models were parallel assignment and single-group assignment, as they represented 48.2% (n=9330) and 44.5% (n=8613) of analyzed studies, respectively. The most common primary purpose of the included studies was treatment, accounting for 66.2% (n=12,815) of cases, followed by supportive care with 10.6% (n=2058). Among observational studies, the most prevalent observational model was cohorts, representing 64% (n=3295) of the studies included in the analysis. In addition, 72.3% (n=3295) of observational studies were prospective, making this more frequently used than perspective.

**Table 2:** Description of the characteristics of oncology studies according to their use of PROMs or PREMs classified by artificial intelligence–enriched algorithm, ClinicalTrials.gov database, 2012-2022 (N=24,491).

Characteristics					All (N=24,491)			Studies not using a PROM^a^ or PREM^b^ (n=16,462)			Studies that used PROMs or PREMs (n=8029)			*P* value	
**Study type**															<.001
	Interventional			19,344 (79)			12,544 (76.2)			6800 (84.7)					
	Observational			5147 (21)			3918 (23.8)			1229 (15.3)					
**Interventional studies**					19,344			12,544			6800			—^c^	
	**Clinical phase**													<.001^d^	
		Early phase 1	376 (1.9)			314 (2.5)			62 (0.9)						
		Phase 1	2536 (13.1)			2253 (18)			283 (4.2)						
		Phase 1/2	1528 (7.9)			1279 (10.2)			249 (3.7)						
		Phase 2	5228 (27)			3905 (31.1)			1323 (19.5)						
		Phase 2/3	289 (1.5)			165 (1.3)			124 (1.8)						
		Phase 3	1650 (8.5)			836 (6.7)			814 (12)						
		Phase 4	452 (2.3)			274 (2.2)			178 (2.6)						
		Missing data^e^	7285 (37.7)			3,518 (28)			3767 (55.4)						
	**Allocation type**													<.001^d^	
		Nonrandomized	2543 (13.1)			2025 (16.1)			518 (7.6)						
		Randomized	8586 (44.4)			4185 (33.4)			4401 (64.7)						
		NA^f^	8215 (42.5)			6334 (50.5)			1881 (27.7)						
	**Interventional model**													<.001^d^	
		Crossover assignment	374 (1.9)			206 (1.6)			168 (2.5)						
		Factorial assignment	120 (0.6)			58 (0.5)			62 (0.9)						
		Parallel assignment	9330 (48.2)			4910 (39.1)			420 (65)						
		Sequential assignment	873 (4.5)			725 (5.8)			148 (2.2)						
		Single group assignment	—			6619 (52.8)			1994 (29.3)						
		Missing data	34 (0.2)			26 (0.2)			8 (0.1)						
	**Primary purpose**													<.001^d^	
		Diagnostic	1573 (8.1)			1401 (11.1)			172 (2.5)						
		Prevention	1031 (5.3)			561 (4.5)			470 (6.9)						
		Supportive care	2058 (10.6)			420 (3.3)			1638 (24.1)						
		Treatment	12,815 (66.2)			9026 (72)			3789 (55.7)						
		Other	1787 (9.2)			1,082 (8.6)			705 (10.4)						
		Missing data	80 (0.4)			54 (0.4)			26 (0.4)						
**Observational studies**					5147			3918			1229			—	
	**Observational model**													<.001^d^	
		Case-control	535 (10.4)			441 (11.3)			94 (7.6)						
		Case-only	736 (14.3)			580 (14.8)			156 (12.7)						
		Cohort	3295 (64)			2460 (62.8)			835 (67.9)						
		Other	521 (10.1)			396 (10.1)			125 (10.2)						
		Missing data	60 (1.2)			41 (1)			19 (1.5)						
	**Time perspective**													<.001^d^	
		Cross-sectional	359 (7)			243 (6.2)			116 (9.4)						
		Prospective	3723 (72.3)			2742 (70)			981 (79.8)						
		Retrospective	836 (16.2)			753 (19.2)			83 (6.8)						
		Other	204 (4)			161 (4.1)			43 (3.5)						
		Missing data	25 (0.5)			19 (0.5)			6 (0.5)						

^a^PROM: patient-reported outcomes measure.

^b^PREM: patient-reported experience measure.

^c^Not available.

^d^*P* values do not account for missing data, as the data are censored.

^e^Missing data does not correspond to any clinical trial phase as per its definition (eg, medical devices).

^f^Trials classified as single group assignment and sequential assignment are grouped under “NA” for allocation type.

Table 2 presents the distribution of key study characteristics for all included studies (N=24,491), stratified by whether they used patient-reported outcome measures (PROMs) or patient-reported experience measures (PREMs). Characteristics are shown separately for interventional and observational studies, with proportions compared between studies using and not using PROMs or PREMs. Significant differences were found across study type, clinical phase, allocation method, interventional model, primary purpose, observational model, and time perspective (all *P*<.001). MCAR test using the RBtest R package indicated that data on the clinical phase were missing at random, while data for the interventional model, primary purpose, observational model, and time perspectives were missing completely at random.

Among all studies included in the analysis, 32.8% (n=8029) incorporated at least one PROM or PREM, as identified by the AI-enriched algorithm. Out of those, 84.7% (n=6800) were interventional, while 15.3% (n=1229) were observational. Among interventional studies, phase 2 and phase 3 trials incorporated mostly PROMs and PREMs, representing 19.5% (n=1323) and 12.0% (n=814) of them, respectively. Nevertheless, 55.4% (n=3767) of interventional studies did not provide any exploitable data on their clinical phases. Interventional studies incorporating PROMs and PREMs were mainly randomized (n=4401, 64.7%). The main interventional models were mainly parallel assignment and single-group assignment, as they represented 65% (n=4420) and 29.3% (n=1994), respectively, of studies with PROMs or PREMs. Treatment (n=3789, 55.7%) and supportive care (n=1638, 24.1%) were the most frequent primary purposes of interventional studies using PROMs or PREMs. Among observational studies using PROMs or PREMs (n=1229), 67.9% (n=835) were cohort studies, while 12.7% (n=156) were case-only studies. Considering time perspective, prospective studies were the most frequent to incorporate PROMs or PREMs, representing 79.8% (n=981) of them.

### PROMs and PREMs Use by Sites of Cancer

Proportions of organ-specific oncology studies using at least one PROM or PREM are shown in Figure S3 in Multimedia Appendix 1. Breast and digestive cancers accounted for nearly 41% (2818/6918) of all studies using PROMs or PREMs in oncology. Urogenital, lung, and prostate cancers followed, representing 8% (579/6918), 10% (673/6918), and 11% (756/6918), respectively, of all studies using PROMs or PREMs in oncology. Proportions of organ-specific oncology studies using at least one PROM or PREM, as identified by the AI-enriched algorithm, are shown in Figure S3 in Multimedia Appendix 1.

### PROMs and PREMs Used in Oncology Studies

Among the 8029 studies that included at least one PROM or PREM, as identified by the AI-enriched algorithm, 4823 (61.1%) used one or more of the 9 most commonly used PROMs or PREMs. Among these, the EORTC QLQ-C30 was the most frequently used questionnaire among PROMs and PREMs in clinical studies in oncology. It was used in approximately 33.2% (n=1602) of the studies included in our analysis. The FACT-G and the EQ-5D-5L questionnaires were the next most frequently used PROMs or PREMs, appearing in 15.2% (n=734) and 11.5% (n=552) of the studies analyzed, respectively (Table 3). Table 4 presents the categories of health dimensions assessed by the instruments identified across the 4823 studies that used one or more of the 9 most commonly used PROMs or PREMs, as identified by the AI-enriched algorithm. In 40.7% (n=1969) of the identified cases, the PROMs or PREMs focused on care-related patient satisfaction, followed by organ-specific quality of life (n=783, 16.2%), as well as depression and anxiety scales (n=441, 9.1%). This distribution results from the zero-shot, multilabel classification model, which often assigned satisfaction in overlap with the quality of life. The latter was further divided into general and cancer-specific subcategories. No additional filters based on study type or trial phase were applied to obtain these proportions.

**Table 3:** Instruments repartition among oncology studies that used one or more of the nine most commonly used patient-reported outcome measures or patient-reported experience measures, as identified by an artificial intelligence–enriched algorithm, ClinicalTrials.gov database, 2012-2022 (n=4823)^a^.

Instruments	Value and proportion, n (%)
EORTC QLQ-C30^b^	1602 (33.2)
FACT-G^c^	734 (15.2)
Cancer‑Related QoL^d^	416 (8.6)
HADS^e^	441 (9.1)
C-VAS^f^	263 (5.5)
NCI^g^	411 (8.5)
EQ-5D-5L	552 (11.5)
PRO-CTCAE^h^	292 (6.1)
IPSS^i^	112 (2.3)

^a^This table summarizes the frequency and proportion of studies that used at least one of the nine most frequently used patient-reported outcome measures (PROMs) or patient-reported experience measures (PREMs). The most commonly used instrument was the EORTC QLQ-C30 (33.2%), followed by FACT-G (15.2%) and EQ-5D-5L (11.5%). Less frequently used instruments included IPSS and C-VAS.

^b^EORTC QLQ-C30: European Organisation for Research and Treatment of Cancer Quality of Life Questionnaire-Core 30.

^c^FACT-G: Functional Assessment of Cancer Therapy – General.

^d^Cancer Related QoL: Cancer-Related Quality of Life.

^e^HADS: Hospital Anxiety and Depression Scale.

^f^C-VAS: Cancer Visual Analogue Scale.

^g^NCI: National Cancer Institute.

^h^PRO-CTCAE: Patient-Reported Outcomes version of the Common Terminology Criteria for Adverse Events.

^i^IPSS: International Prostate Symptom Score.

**Table 4:** Categories of Health Dimensions Assessed by Instruments Identified among oncology studies that used one or more of the 9 most commonly used PROMs or PREMs, as identified by AI-enriched algorithm, ClinicalTrials.gov database, 2012-2022 (n=4823)^a^.

Categories	Values and proportion, n (%)
Patient satisfaction with care	1969 (40.7)
Organ-specific quality of life	783 (16.2)
Depression and anxiety scale	441 (9.1)
NA	1640 (33.9)

^a^This table shows the distribution of health dimensions evaluated by instruments used in studies using one or more of the nine most frequently used PROMs and PREMs. The majority of instruments assessed patient satisfaction with care (40.7%), followed by organ-specific quality of life (16.2%) and depression and anxiety scales (9.1%). A notable proportion (33.9%) of assessments were classified as not applicable (NA).

Figure S4 in Multimedia Appendix 1 provides the distribution of the various scales commonly used in identified clinical studies, categorized by cancer site. Breast and digestive cancers were the most represented in the use of PROMs or PREMs in oncology studies, accounting for 22% (1521/6918) and 20% (1307/6918) of cases, respectively. These were followed by prostate (756/6918, 11%), lung (673/6918, 10%), urogenital (579/6918, 8%), and head and neck cancers (408/6918, 6%). In contrast, hematopoietic (193/6918, 3%), brain (118/6918, 3%), thyroid (57/6918, 1%), skin (52/6918, 1%), bone (118/6918, 1%), and neuroendocrine cancers (24/6918, 0.4%) were less frequently studied using PROMs or PREMs.

The use of PROMs and PREMs, as identified by the AI-enriched algorithm, varied according to several characteristics of studies (Figure S5A in Multimedia Appendix 1). Out of interventional studies, studies in phases 2 to 4 were incorporating PROMs or PREMs more often than preclinic or early phase 1 studies (OR 1.8, 95% CI 1.1-2.8 for phase 2; OR 3.6, 95% CI 2 .3-5.8 for phase 3; OR 2.6, 95% CI 1.6-4.4 for phase 4). Randomized clinical trials used twice as many PROMs or PREMs as nonrandomized trials (OR 2, 95% CI 1.7-2.4). Clinical trials focusing on supportive care demonstrated significantly higher odds for using PROMs or PREMs than trials with other objectives (OR 4.1, 95% CI 2.7-6.2). Studies concerning some organ-specific trials were less likely to use PROMs or PREMs than non–organ-specific ones—such as breast (OR 0.7, 95% CI 0.5-0.9), digestive (OR 0.6, 95% CI 0.5-0.8), lung (OR 0.7, 95% CI 0.4-0.7), hemopoietic (OR 0.5, 95% CI 0.3-0.8), and thyroid (OR 0.4, 95% CI 0.2-0.9) cancers. Similarly, the use of PROMs or PREMs instruments in observational studies differed according to characteristics of studies (Figure S5B in Multimedia Appendix 1). In contrast to retrospective studies, cross-sectional and prospective studies used PROMs or PREMs more often (OR 4.6, 95% CI 3.3-6.4 and OR 3.2, 95% CI 2.5-4.1, respectively). After adjusting for confounding factors, cohort studies had significantly higher odds for using PROMs or PREMs than case-control studies (OR 1.6, 95% CI 1.3-2.1). Additionally, studies focusing on organ-specific trials, such as breast (OR 0.7, 95% CI 0.5-0.8), digestive (OR 0.4, 95% CI 0.3-0.5), head and neck (OR 0.6, 95% CI 0.4-0.8), hematopoietic (OR 0.6, 95% CI 0.4-0.8), lung (OR 0.4, 95% CI 0.3-0.5), thyroid (OR 0.5, 95% CI 0.3-0.9), and urogenital (OR 0.4, 95% CI 0.3-0.6) cancers, had lower odds for using PROMs or PREMs than non-organ-specific studies.

## Discussion

### Main Study Findings

This observational study focused on the ability of an AI-based tool to identify whether or not PROMs or PREMs were used in oncology trials from ClinicalTrials.gov, as compared to an expert-based gold standard. Taken together, our results on 24,491 studies clearly show that (1) an AI-enriched algorithm outperformed an expert-based one in identifying PROMs or PREMs in clinical studies, (2) the use of PROMs or PREMs has increased over time, (3) studies in breast cancer and digestive cancer accounted for nearly half of the studies incorporating PROMs or PREMs, and (4) interventional prospective studies were more likely to use PROMs or PREMs than any other type of studies.

### Comparison With Prior Work and Implications

The AI-enriched algorithm that we developed for this specific purpose appears particularly promising for researchers seeking to identify both the presence of PROMs or PREMs and which PROM or PREM was used in oncology studies. With a 90% accuracy, we were able to better identify studies than experts would do manually and in a more efficient way: first, the manual checking of 4770 outcomes (accounting for 890 studies) took several weeks using a work force of 16 high-skilled people, while the algorithm was able to classify the whole 24,491 studies within a few minutes (from 10 minutes to 3 hours, depending on the GPU workforce). Although the development of the AI-enriched algorithm required a considerable initial investment in time and expertise, this effort represents a long-term return on investment, as the tool can subsequently process large datasets much faster than expert-based approaches. A significant increase in the use of PROMs or PREMs in phase 2 to 4 studies could be forecasted in the coming years, driven by the growing interest from key stakeholders, including industry, regulatory bodies, patients, and patient associations, as well as their commitment to promoting value-based health care (VBHC) in clinical practice. There is a significant and growing interest in the use of these tools across clinical research. Recently, regulatory agencies, such as the FDA and HAS, encourage the use of PROMs or PREMs when appropriate and have incorporated them into care quality and safety indicators, but their inclusion in clinical trials is not mandatory [19-21]. Additionally, the increasing incorporation of PROMs or PREMs in clinical studies that we report supports our optimism. The AI-enriched algorithm tool could be extended to other medical indications, fostering widespread research on PCM. On a larger scale, AI could become an invaluable tool for medical stakeholders to manage, conduct, and monitor clinical studies [27]. Nevertheless, the AI-enriched method also carries the risk of misclassifying conceptually close types of clinical outcome assessments (eg, observer-reported outcomes or clinician-reported outcomes) as PROMs or PREMs, potentially leading to an overestimation of their prevalence compared with the expert-based review. In contrast, the expert-based review offers the advantage of manual verification by trained reviewers, which helps ensure accuracy and contextual understanding, even if it is more resource-intensive.

### New Findings in the Field of PROMs and PREMs

Among all organ-specific studies, PROMs and PREMs were more frequently used in studies focusing on breast and digestive cancers. This trend may be attributed to the extensive research on these cancers, driven by their high prevalence, significant impact on patients’ quality of life, and the growing need for patient feedback as treatments evolve [28,29]. Furthermore, the diverse patient populations affected by breast and digestive cancers, spanning various age groups, genders, and ethnic backgrounds, underscore the importance of considering patients’ perceived experiences and outcomes. While this is not unique to these cancer sites, breast and digestive cancers are among the most extensively studied in terms of patient-reported outcomes [30,31], likely due to their high prevalence and the strong presence of advocacy groups and research funding. This visibility has facilitated the integration of VBHC principles, where patient-centered care remains a central theme in medical research [32]. Compared to other cancer sites, breast and digestive cancers feature more prominently in VBHC-related literature, highlighting a gap that should be addressed for less-studied malignancies. It is also possible that in therapeutic areas where PROMs and PREMs are less common or less standardized, the relative performance gap between AI- and expert-based methods could be smaller, underscoring the importance of context when interpreting these findings. In our dataset, where PROMs and PREMs were present in 33% of oncology studies, the algorithm achieved strong sensitivity and specificity; however, because sensitivity and specificity depend on the prevalence of the measured outcome, its performance may be reduced in contexts where PROMs and PREMs are less frequently or less consistently reported. Further studies are therefore needed to assess the robustness and generalizability of AI-driven approaches across diverse conditions beyond the oncology field.

We also report that interventional or prospective studies incorporated more PROMs or PREMs than observational or retrospective studies. This trend could be attributed to the growing interest of regulatory bodies and sponsors (eg, industry, investigators, and promoters) in incorporating these measures into clinical trials, including regulators, such as the FDA and European Medicines Agency, as well as Health Technology Assessment bodies, in supporting approvals and decision-making. In such cases, some governments have begun recommending the use of PROMs or PREMs in interventional studies through official communications and guidelines [20,33]. Interventional studies are, by definition, designed with a structured protocol that allows for the systematic collection of specific data, making them particularly suited for incorporating PROMs or PREMs. Furthermore, since PROMs or PREMs provide direct patient insights, their inclusion in interventional studies is especially relevant, as these studies aim to evaluate the effects of specific treatments or interventions. Incorporating PROMs or PREMs into the design of observational studies requires their integration into routine care [34]. Another approach would involve leveraging PCMs in registries or other real-world data sources and incentivizing the use of PROMs or PREMs in funding and publishing by updating publication guidelines and funding requirements. Collaborative approaches, such as engaging patient advocacy groups and fostering multidisciplinary teams, may further enhance their adoption, as these groups often emphasize holistic, patient-centered care and bring together diverse clinical perspectives that recognize the value of patient-reported measures [35,36].

### Strengths and Limitations

To address potential sources of bias, several measures were implemented. Selection bias was minimized by including all eligible oncology studies registered in ClinicalTrials.gov rather than a sample. Information bias related to outcome misclassification was reduced using a double-reference approach, where the AI-enriched algorithm was trained and evaluated against an expert-consensus gold standard. Misclassification due to heterogeneous naming of PROMs and PREMs was partially mitigated through expert review and consensus labeling during model development. Finally, analytical bias was limited by applying the same preprocessing pipeline and evaluation metrics across all studies. Additionally, the absence of prespecified subgroups or sensitivity analyses may limit the exploration of residual heterogeneity.

One important limitation of our AI-based tool is its risk of false positive or false negative identification of PROMs or PREMs. The model sometimes struggles with multiword PROMs or PREMs (see Multimedia Appendix 1 for examples). Even when a full name like the “Short Form 36 Health Survey” is right there in the text, the AI might break it into pieces, tagging “Short Form” and “Health Survey” as separate things instead of recognizing them as the single, standardized SF-36, which leads to the AI algorithm not recognizing it as a PROM. Moreover, uppercase abbreviations can be a “red herring” for the model. Since many PROMs and PREMs are abbreviated in all caps, the AI occasionally misidentifies general clinical terms (like “PAIN,” which is a symptom) as a PROM or PREM.

The increased use of PROMs and PREMs in oncology was also earlier reported by Vodicka et al [37], who aimed to estimate the proportion of clinical trials using at least one PROM or PREM from November 2007 to December 2013 [37]. They identified 29% of oncology trials using PROMs or PREMs, where we identified 33%. This could either be due to an improvement in the method for identifying studies or to an increase in the use of PROMs and PREMs over time. We used the same ClinicalTrials.gov database with a larger number of studies. The main difficulty in identifying PROMs and PREMs lay in the classification used in the ClinicalTrials.gov database. No standardized naming for PROMs and PREMs was currently available, leading researchers and HCPs to use the same PROMs or PREMs but with different names. This observation may explain the poor quality of data retrieved from studies used by our AI-enriched algorithm. Both algorithms (AI-enriched and expert-based ones) exhibited the same issue, but our AI-enriched algorithm remained more efficient than the expert-based one. Therefore, classification appeared to be the major risk of bias in our study. This risk of bias was not assessed in this study: future studies should test its clinical use, including whether the 10% of errors of this tool would be clinically meaningful or not. Both the trends in the use of PROMs and PREMs in oncology trials over time and their distribution exhibited performance rates below 100%, due to misclassification of cancers. One potential way to reduce this bias would be the implementation of an AI-based tool to support researchers in identifying, labeling, and naming PROs, thereby enhancing consistency in classification, terminology (names), and acronyms. Our study also experienced a limitation related to the dataset we used, especially regarding (1) the heterogeneity of the registries included, (2) the reliance on English-language parsing tools, and (3) the fact that our approach did not involve full-text scrutiny. Another limitation of our study is the lack of external validation; as we used the overall population of studies in the field of oncology from the ClinicalTrials.gov database, this would require the use of another dataset, either emerging from another field than oncology or from another database than ClinicalTrials.gov, for evaluating the generalizability of our findings. Such a study should also involve a large dataset. Finally, we chose to compare our AI-based algorithm to an expert-based algorithm against a human-based gold standard. This did not include any comparison with other algorithms using any machine learning tool, which is also a limitation. Future algorithms developed for this purpose may be tested against the one we report here.

Overall, within the oncology field, evidence suggests that routine collection of PROMs and PREMs enhances patient-provider communication, helps identify symptoms and/or concerns, and improves patient satisfaction [38]. However, there is currently limited evidence demonstrating their impact in oncological settings, particularly regarding an effective improvement of quality or a better health care system performance [39,40]. Therefore, further research is essential to address these gaps, assess the applicability of this approach across a broader range of pathologies, and evaluate its adaptability to additional open-access databases. Such investigations would help determine the generalizability and scalability of the methodology, facilitating its integration into diverse health care data systems and supporting its potential for widespread application in health data analysis and patient-centered research.

Importantly, this study is innovative because it combines expert-labeled data with artificial intelligence to automate the identification of PROMs and PREMs, which overcomes the limitations of previous approaches that relied solely on manual review or keyword-based searches. Unlike prior studies, our AI-enriched method allows large-scale, reproducible monitoring of patient-reported measures across thousands of trials, providing a clear contribution to the field by quantifying trends, highlighting gaps, and identifying areas for improvement in patient-centered data collection.

This approach can support researchers, regulators, sponsors, and health care systems in better designing trials, in more easily tracking the integration of PROMs and PREMs, in better informing funding and policy decisions, and ultimately in promoting value-based, patient-centered care.

### Conclusions

In conclusion, our data clearly demonstrate that an AI-enriched algorithm is more accurate, in terms of sensitivity and specificity, and faster than traditional expert-based methods for identifying PROMs and PREMs in oncology clinical studies. PROMs and PREMs usage is increasing over time, particularly in interventional and prospective studies. This approach is innovative because it combines expert-labeled data with AI, enabling scalable and reproducible identification of PROMs and PREMs, unlike previous manual or expert-based methods. We suggest that improving the naming and classification of PROMs and PREMs could further facilitate their identification and use in studies, for example, through the development of an AI-based tool to assist researchers in identifying, labeling, and naming patient-reported outcomes. Future studies should extend this methodology to other pathologies and databases, enhancing its use and supporting broader integration of patient-reported measures in clinical research.

## Data Availability

All databases are available upon reasonable request to the corresponding author.

## Appendix

Multimedia Appendix 1 STROBE checklist, supplementary figures on patient-reported outcome measure and patient-reported experience measure use in oncology (2012–2022), and missing error analysis.

## Abbreviations

AI: artificial intelligence
BERT: bidirectional encoder representations from transformers
EORTC: European Organization for Research and Treatment of Cancer
FDA: US Food and Drug Administration
FSS: Fatigue Severity Scale
HAS: Haute Autorité de Santé - French Authority of Health
HCP: health care provider
MCAR: missing completely at random
NER: named entity recognition
OR: odds ratio
PCM: patient-centered measure
PREM: patient-reported experience measure
PROM: patient-reported outcome measure
PROQOLID: Patient-Reported Outcome and Quality of Life Instruments Database
QLQ: Quality of Life Questionnaire
QLQ-C30: Quality of Life Questionnaire-Core 30
STROBE: Strengthening the Reporting of Observational Studies in Epidemiology
VBHC: value-based health care
