# Supplementary material for: Artificial Intelligence for Identifying Patient-Reported Outcome and Experience Measures in Oncology: Retrospective Cross-Sectional Study Using ClinicalTrials.gov
Source: J Med Internet Res. 2026 Apr 16;28:e84533. doi: 10.2196/84533 (PMC13133595; doi:10.2196/84533)
Supplement: Multimedia Appendix 1 [file jmir_v28i1e84533_app1.docx]

***Title***

*Artificial Intelligence (AI)-powered Expertise: a real asset for identifying patient-reported measurements (PROMs and PREMs) in clinical studies.*

***Authors***

Jessica Soyer^1^*, MSc, Akram Hecini^1^*, MSc, Sylvain Juchet^1^, MSc, Céline Desvignes-Gleizes^2^, PhD, Maxime Thiebaut^1^, PharmD, MSc, Jean-Philippe Bertocchio^1,3,4,5^, MD, PhD

* contributed equally to this work

***Affiliations***

^1^ SKEZI, Les Papèteries – Image Factory, 1 Esplanade Augustin Aussédat, 74960 Annecy, France

^2^ Mapi Research Trust, 27 rue de la Villette, 69003 Lyon, France

^3^ Thyroïde – Tumeurs Endocrines, Pitié-Salpêtrière Hospital, Assistance Publique-Hôpitaux de Paris, Boulevard de l’hôpital, 75013 Paris, France

^4^ Reference center for rare diseases related to calcium-phosphate metabolism disorders, Pitié-Salpêtrière Hospital, Assistance Publique-Hôpitaux de Paris, Boulevard de l’hôpital, 75013 Paris, France

^5^ Université Paris Cité, INSERM U1333 Santé Orale, FHU-DDS-net, Dental School, Montrouge, France

***Correspondance***

Jean-Philippe Bertocchio: [jean-philippe.bertocchio@skezi.eu](mailto:jean-philippe.bertocchio@skezi.eu)

***Contributions***

JS performed analysis, wrote first draft of the manuscript, revised the manuscript.

AH performed analysis, wrote first draft of the manuscript, revised the manuscript.

SJ designed the study, revised the manuscript.

CDG provided the list of PROMs/PREMs-related terms using the PROQOLID™ (Mapi Research Trust) database.

MT performed the analysis and the data layout, wrote further drafts of the manuscript and the appendix, revised the manuscript.

JPB designed the study, supervised the study, wrote the first draft of the manuscript, revised the manuscript.

**Data availability**

Data will be made available upon reasonable request to the corresponding author.

**Conflict of interest disclosure**

SJ, HA, MT and JPB work at SKEZI, a digital platform that develops tools for collecting patient-reported outcomes. CDG works for a company that protects PROMs/PREMs.

Summary

[STROBE Statement Checklist 3](#_Toc221527478)

[Figure 2: Trends in the use of PROMs/PREMs in oncology studies, as identified by AI-enriched algorithm, ClinicalTrial.gov database, 2012-2022 (n = 8,029) 6](#_Toc221527479)

[Figure 3: Distribution of the use of PROMs/PREMs in oncology studies by site of cancer, as identified by AI-enriched algorithm, ClinicalTrial.gov database, 2012-2022 (n = 8,029) 7](#_Toc221527480)

[Figure 4: Sankey diagram illustrating relationship between sites of cancers and concepts measured by the most used PRO, as identified by AI-enriched algorithm, ClinicalTrial.gov database, 2012-2022 (n = 4,823) 8](#_Toc221527481)

[Figure 5: Factors associated with the use of PROMs/PREMs in interventional studies, as identified by AI-enriched algorithm, ClinicalTrial.gov database, 2012-2022 (n = 19,344) 9](#_Toc221527482)

[Figure 6: Factors associated with the use of PROMs/PREMs in observational studies, as identified by AI-enriched algorithm, ClinicalTrial.gov database, 2012-2022 (n = 5,147) 10](#_Toc221527483)

[Table 1: Missing Error Analysis: 10](#_Toc221527484)

# STROBE Statement Checklist

|  | **Item No** | **Recommendation** | **Page No** |
| --- | --- | --- | --- |
| **Title and abstract** | 1 | (*a*) Indicate the study’s design with a commonly used term in the title or the abstract | 1-3 |
|  |  | (*b*) Provide in the abstract an informative and balanced summary of what was done and what was found | 2-3 |
| **Introduction** | | | |
| Background/rationale | 2 | Explain the scientific background and rationale for the investigation being reported | 4 |
| Objectives | 3 | State specific objectives, including any prespecified hypotheses | 5 |
| **Methods** | | | |
| Study design | 4 | Present key elements of study design early in the paper | 6 |
| Setting | 5 | Describe the setting, locations, and relevant dates, including periods of recruitment, exposure, follow-up, and data collection | 6 |
| Participants | 6 | (*a*) Give the eligibility criteria, and the sources and methods of selection of participants | 6 |
| Variables | 7 | Clearly define all outcomes, exposures, predictors, potential confounders, and effect modifiers. Give diagnostic criteria, if applicable | 6-9 |
| Data sources/ measurement | 8* | For each variable of interest, give sources of data and details of methods of assessment (measurement). Describe comparability of assessment methods if there is more than one group | 6-9 |
| Bias | 9 | Describe any efforts to address potential sources of bias | 6-9 |
| Study size | 10 | Explain how the study size was arrived at | 24 |
| Quantitative variables | 11 | Explain how quantitative variables were handled in the analyses. If applicable, describe which groupings were chosen and why | 9 |
| Statistical methods | 12 | (*a*) Describe all statistical methods, including those used to control for confounding | 8-9 |
|  |  | (*b*) Describe any methods used to examine subgroups and interactions | 8-9 |
|  |  | (*c*) Explain how missing data were addressed | 10 |
|  |  | (*d*) If applicable, describe analytical methods taking account of sampling strategy | 9 |
|  |  | (*e*) Describe any sensitivity analyses | No sensitivity analyses were prespecified or conducted (See p.11) |
| **Results** | | | |
| Participants | 13* | (a) Report numbers of individuals at each stage of study—eg numbers potentially eligible, examined for eligibility, confirmed eligible, included in the study, completing follow-up, and analysed | 24 |
|  |  | (b) Give reasons for non-participation at each stage | This registry-based study used publicly available data and did not involve participant recruitment (See p.6) |
|  |  | (c) Consider use of a flow diagram | 24 |
| Descriptive data | 14* | (a) Give characteristics of study participants (eg demographic, clinical, social) and information on exposures and potential confounders | 26 |
|  |  | (b) Indicate number of participants with missing data for each variable of interest | 26 |
| Outcome data | 15* | Report numbers of outcome events or summary measures | 26 |
| Main results | 16 | (*a*) Give unadjusted estimates and, if applicable, confounder-adjusted estimates and their precision (eg, 95% confidence interval). Make clear which confounders were adjusted for and why they were included | 11 |
|  |  | (*b*) Report category boundaries when continuous variables were categorized | Continuous variables were analyzed as reported and were not categorized (See p.11) |
|  |  | (*c*) If relevant, consider translating estimates of relative risk into absolute risk for a meaningful time period | Results are expressed as odds ratios rather than relative risks (See p.11) |
| Other analyses | 17 | Report other analyses done—eg analyses of subgroups and interactions, and sensitivity analyses | No subgroup, interaction, or sensitivity analyses were prespecified or conducted (See p.11) |
| **Discussion** | | | |
| Key results | 18 | Summarise key results with reference to study objectives | 15 |
| Limitations | 19 | Discuss limitations of the study, taking into account sources of potential bias or imprecision. Discuss both direction and magnitude of any potential bias | 17 |
| Interpretation | 20 | Give a cautious overall interpretation of results considering objectives, limitations, multiplicity of analyses, results from similar studies, and other relevant evidence | 19 |
| Generalisability | 21 | Discuss the generalisability (external validity) of the study results | 18 |
| **Other information** | | | |
| Funding | 22 | Give the source of funding and the role of the funders for the present study and, if applicable, for the original study on which the present article is based | 20 |

*Give information separately for exposed and unexposed groups.

**Note:** An Explanation and Elaboration article discusses each checklist item and gives methodological background and published examples of transparent reporting. The STROBE checklist is best used in conjunction with this article (freely available on the Web sites of PLoS Medicine at http://www.plosmedicine.org/, Annals of Internal Medicine at http://www.annals.org/, and Epidemiology at http://www.epidem.com/). Information on the STROBE Initiative is available at www.strobe-statement.org.

# Figure 2: Trends in the use of PROMs/PREMs in both interventional and observational oncology studies, as identified by AI-enriched algorithm, ClinicalTrial.gov database, 2012-2022 (n = 8,029)


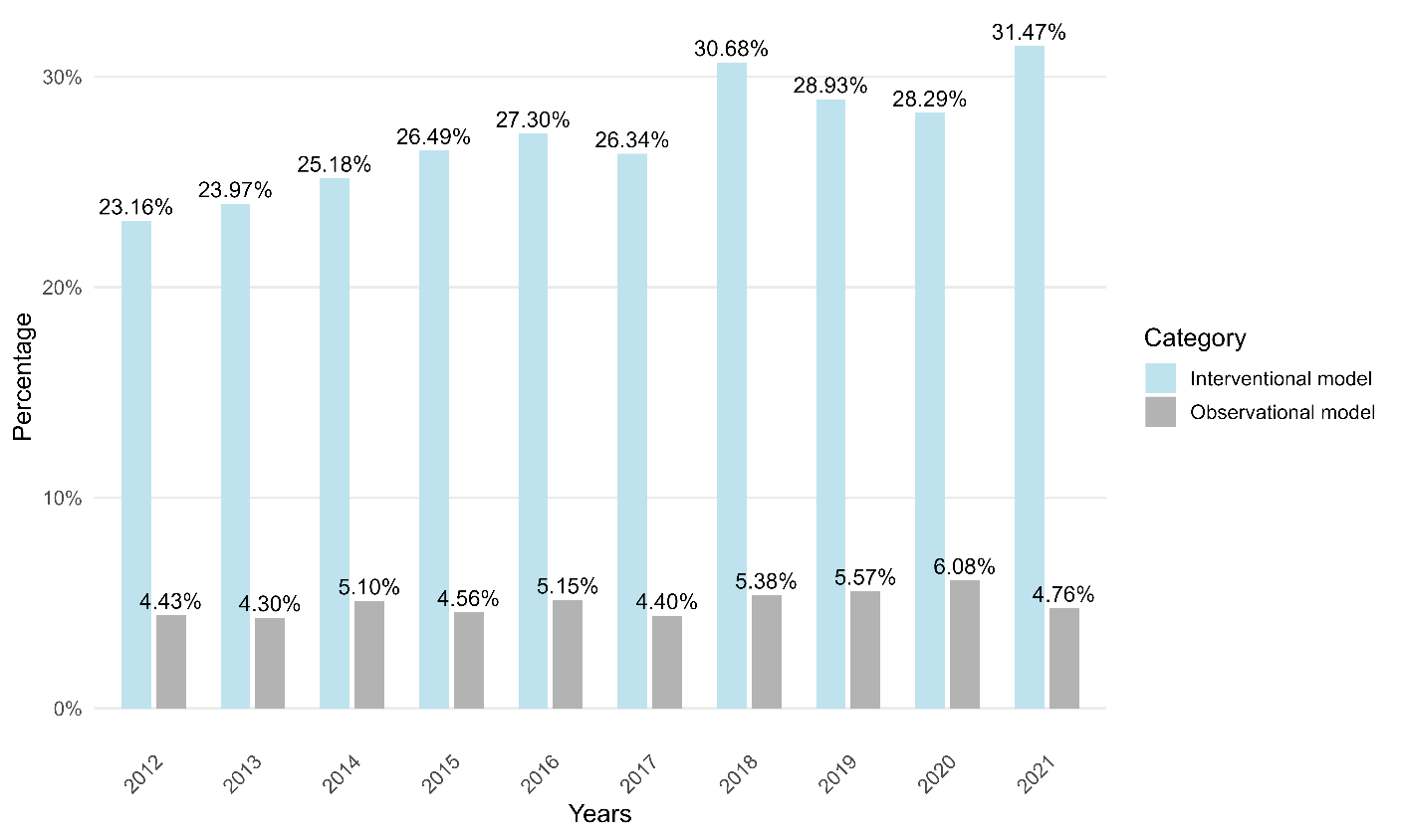


*Abbreviations:* AI: Artificial Intelligence; PREMs: Patient-Reported Outcomes Measures; PROMs: Patient-Reported Outcomes Measures

*Legend:* This figure displays the annual proportion of oncology clinical trials that included Patient-Reported Outcome Measures (PROMs) and/or Patient-Reported Experience Measures (PREMs), as identified by the AI-enriched algorithm, from 2012 to 2021. Bars are divided by study type: interventional trials (blue) and observational trials (grey). The solid line represents the overall trend in PROM/PREM use over time. Data for 2022 were censored due to incomplete extraction at the time of analysis, to avoid bias in proportion estimates; therefore, results are limited to trials registered through December 31, 2021.

# Figure 3: Distribution of the use of PROMs/PREMs in oncology studies by site of cancer, as identified by AI-enriched algorithm, ClinicalTrial.gov database, 2012-2022 (n = 8,029)


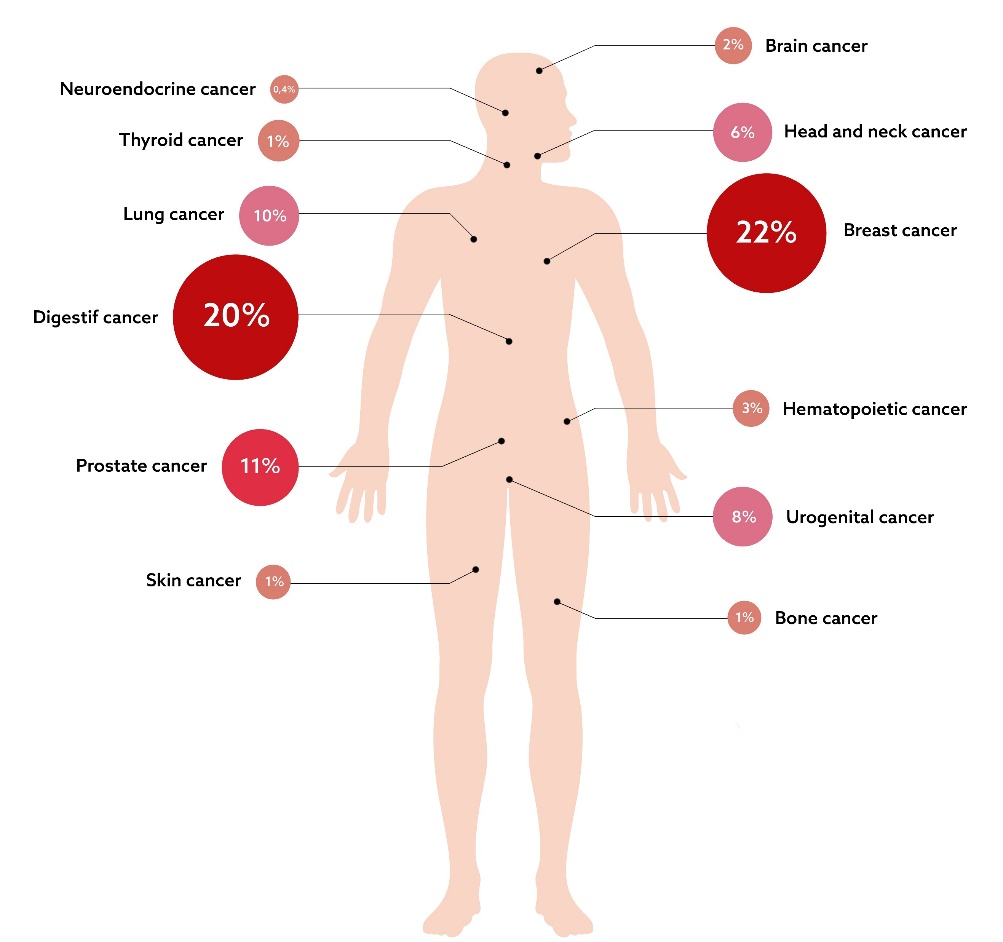


*Abbreviations:* PREMs: Patient-Reported Outcomes Measures; PROMs: Patient-Reported Outcomes Measures

*Legend:* This figure presents the proportion of oncology studies incorporating patient-reported outcome measures (PROMs) and patient-reported experience measures (PREMs) by cancer site. Breast cancer (22%) and digestive cancers (20%) account for the highest usage of PROMs/PREMs, followed by prostate (11%), lung (10%), and urogenital cancers (8%). Lower representation is observed for cancers such as neuroendocrine (0.4%), thyroid (1%), skin (1%), and bone (1%). Circle sizes are scaled to reflect the relative frequency of PROMs/PREMs use across different cancer types.

# Figure 4: Sankey diagram illustrating relationship between sites of cancers and concepts measured by the most used PRO, as identified by AI-enriched algorithm, ClinicalTrial.gov database, 2012-2022 (n = 4,823)


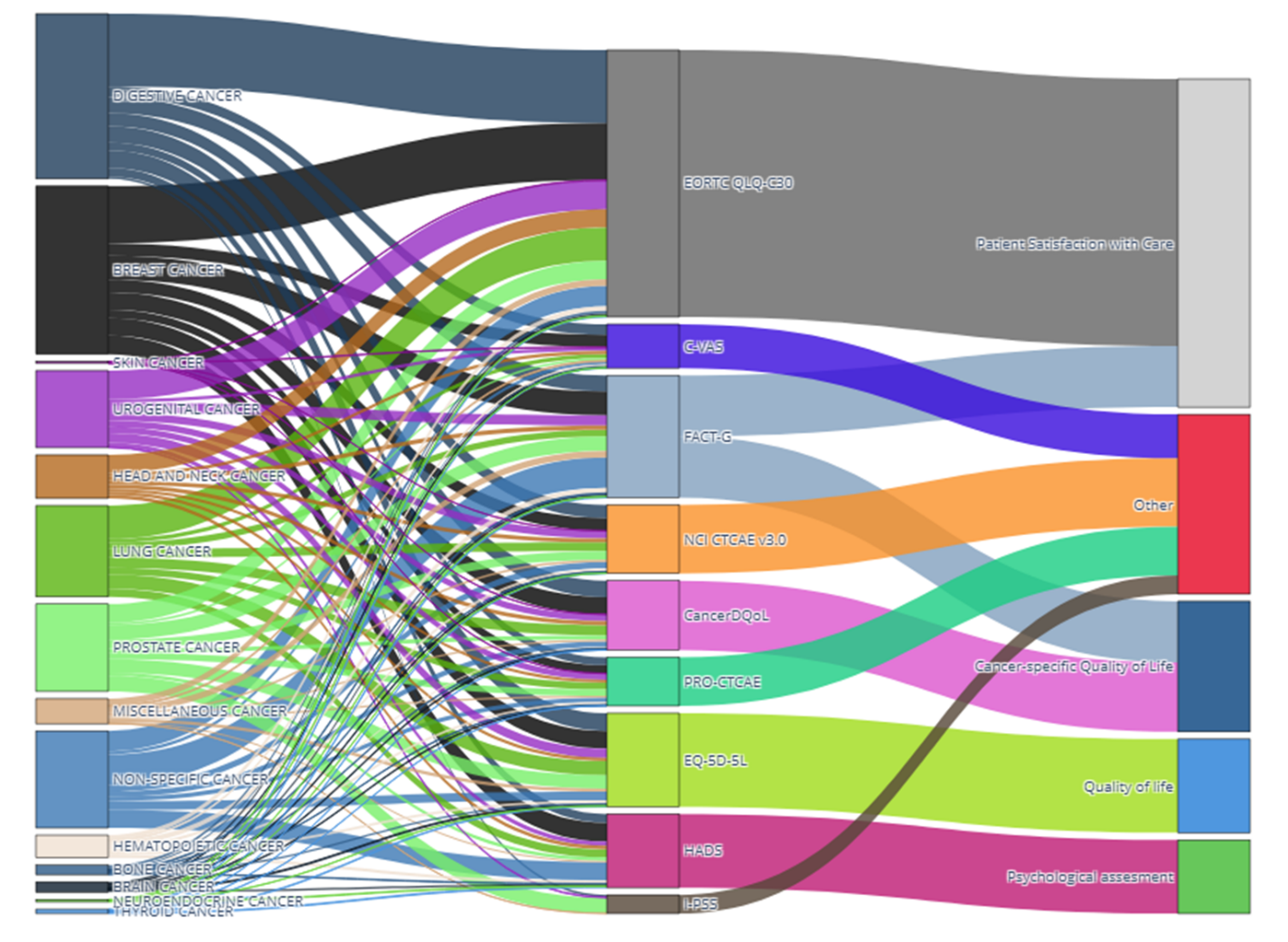


*Abbreviations:* CancerDQoL: Cancer-specific Quality of Life Questionnaire; EORTC QLQ-C30: European Organisation for Research and Treatment of Cancer Quality of Life Questionnaire Core 30; EQ-5D-5L: EuroQol 5 Dimensions 5 Levels; FACT-G: Functional Assessment of Cancer Therapy – General; GVAS: Global Visual Analogue Scale; HADS: Hospital Anxiety and Depression Scale; HPSS: Health Professional Satisfaction Survey; NCI CTCAE v3.0: National Cancer Institute Common Terminology Criteria for Adverse Events version 3.0; PRO: Patient-Reported Outcomes; PRO-CTCAE: Patient-Reported Outcomes version of the Common Terminology Criteria for Adverse Events.

*Legend:* This Sankey diagram visualizes the connections between various cancer sites (left), the most frequently used PRO instruments (center), and the outcome concepts they measure (right) in oncology studies. The EORTC QLQ-C30 emerges as the most widely used instrument, particularly in studies of breast, digestive, and lung cancers, and is primarily associated with the evaluation of patient satisfaction with care. Other commonly employed instruments include FACT-G, EQ-5D-5L, HADS, and PRO-CTCAE, capturing a range of concepts such as general quality of life, cancer-specific quality of life, psychological well-being, and adverse events. The diagram highlights both the diversity of PRO use across cancer types and the conceptual focus of these measures in cancer research.

# Figure 5: Factors associated with the use of PROMs/PREMs in interventional studies, as identified by AI-enriched algorithm, ClinicalTrial.gov database, 2012-2022 (n = 19,344)


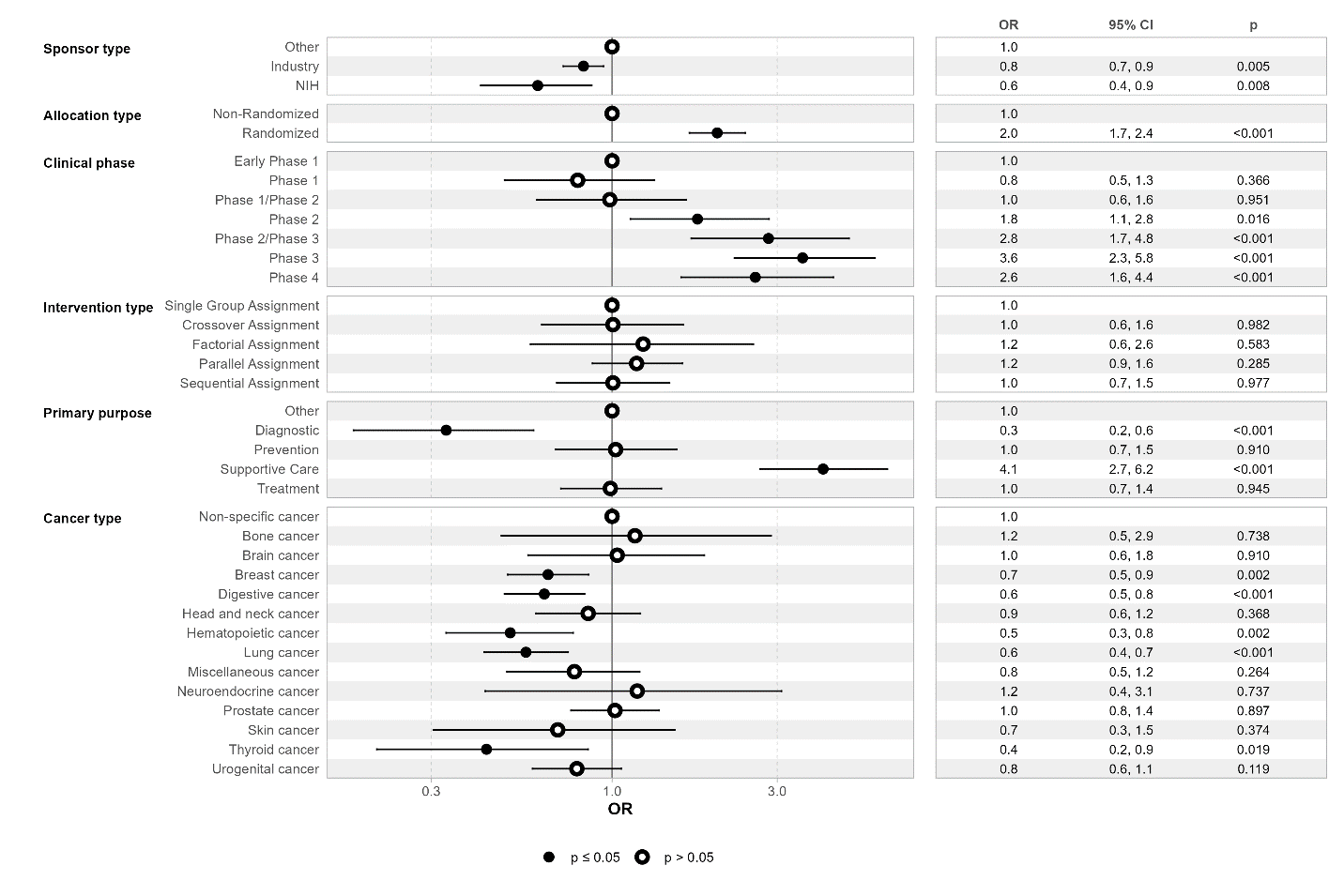


*Abbreviations:* AI: Artificial Intelligence; CI: Confidence Interval; NIH : National institute for Health; OR: Odds ratio

*Legend:* This forest plot displays odds ratios (ORs) with 95% confidence intervals (CIs) for factors associated with the likelihood of utilizing patient-reported outcome measures (PROMs) or patient-reported experience measures (PREMs) in interventional studies employing AI-based algorithms. The analysis includes key study characteristics such as sponsor type, allocation type, clinical phase, intervention type, primary purpose, and cancer type. The vertical dashed line at OR = 1 indicates the reference (no association). Filled circles represent statistically significant associations (p < 0.05), while open circles denote non-significant results (p ≥ 0.05). ORs greater than 1 indicate increased odds of PROMs/PREMs utilization relative to the reference category, while ORs less than 1 indicate decreased odds. Detailed ORs, 95% CIs, and p-values are presented in the accompanying table to the right of the plot.

# Figure 6: Factors associated with the use of PROMs/PREMs in observational studies, as identified by AI-enriched algorithm, ClinicalTrial.gov database, 2012-2022 (n = 5,147)


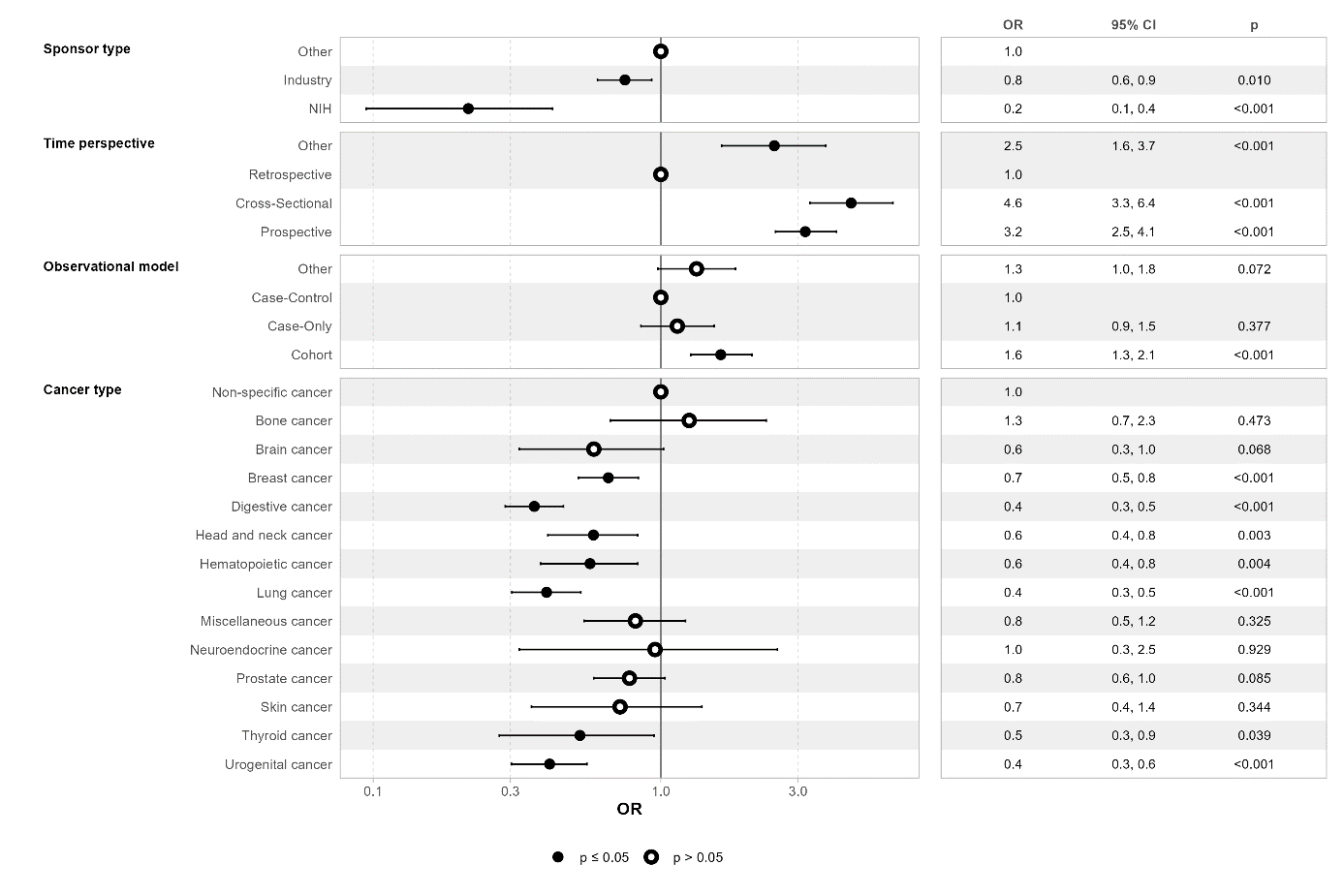


*Abbreviations:* AI: Artificial Intelligence; CI: Confidence Interval; NIH : National institute for Health; OR: Odds ratio

*Legend:* This forest plot presents odds ratios (OR) with 95% confidence intervals (CIs) for factors associated with the likelihood of utilizing patient-reported outcome measures (PROMs) or patient-reported experience measures (PREMs) in observational studies employing AI-based algorithms. Variables assessed include sponsor type, time perspective, observational model, and cancer type. The vertical dashed line at OR = 1 represents the reference value (no association). Filled circles represent statistically significant associations (p < 0.05), while open circles indicate non-significant associations (p ≥ 0.05). ORs greater than 1 indicate increased odds of PROMs/PREMs utilization relative to the reference category, whereas ORs less than 1 indicate decreased odds. Exact OR values, 95% CIs, and p-values are provided in the accompanying table on the right side of the figure.

# Table 1: Missing Error Analysis:

| True PROM name | Model prediction (wrong) | Error type | Missing / incorrect part |
| --- | --- | --- | --- |
| European Organisation for Research and Treatment of Cancer Quality of Life Questionnaire | Organisation for Research and Treatment of Cancer Quality of Life Questionnaire | Missing word at beginning | “European” |
| Short Form Health Survey 36 | Short Form Survey 36 | Missing word in middle | “Health” |
| Hospital Anxiety and Depression Scale | Hospital Anxiety and Depression | Missing word at end | “Scale” |
| EORTC QLQ-C30 | QLQ-C30 | Incomplete abbreviation | “EORTC” |
| SF-36 (Short Form Health Survey 36) | SF / 36 | Fragmented abbreviation | Hyphenated abbreviation split |
